# Long-Term Complications Related to Cardiac Implantable Electronic Devices

**DOI:** 10.3390/jcm14062058

**Published:** 2025-03-18

**Authors:** Jamie Simpson, Mason Yoder, Nathaniel Christian-Miller, Heather Wheat, Boldizsar Kovacs, Ryan Cunnane, Michael Ghannam, Jackson J. Liang

**Affiliations:** 1 Department of Internal Medicine, University of Michigan, Ann Arbor, MI 48109, USA; jamiesim@med.umich.edu (J.S.); masonyo@med.umich.edu (M.Y.); chnathan@med.umich.edu (N.C.-M.); 2 Department of Clinical Electrophysiology, Frankel Cardiovascular Center, University of Michigan, Ann Arbor, MI 48109, USA; hwheat@med.umich.edu (H.W.); boldizk@med.umich.edu (B.K.); rcunnane@med.umich.edu (R.C.); mousajab@med.umich.edu (M.G.)

**Keywords:** cardiac implantable electronic devices, CIED, long-term complications, lead-related complications, CIED-related arrhythmias, CIED infection

## Abstract

Cardiac implantable electronic devices (CIEDs) are commonly used for a number of cardiac-related conditions, and it is estimated that over 300,000 CIEDs are placed annually in the US. With advances in technology surrounding these devices and expanding indications, CIEDs can remain implanted in patients for long periods of time. Although the safety profile of these devices has improved over time, both the incidence and prevalence of long-term complications are expected to increase. This review highlights pertinent long-term complications of CIEDs, including lead-related issues, device-related arrhythmias, inappropriate device therapies, and device-related infections. We also explore key clinical aspects of each complication, including common presentations, patient-specific and non-modifiable risk factors, diagnostic evaluation, and recommended management strategies. Our goal is to help spread awareness of CIED-related complications and to empower physicians to manage them effectively.

## 1. Introduction

Since the first successful pacemaker implantation in 1958, the number of patients with cardiac implantable electronic devices (CIEDs) and the indications for such devices have increased significantly. CIED is a general term used to encompass several different devices, including permanent pacemakers (PPMs), implantable cardioverter–defibrillators (ICDs), cardiac resynchronization therapy (CRT), and implantable or insertable cardiac monitors (also called loop recorders; ILRs) [1]. In the United States alone, it is estimated that over 300,000 CIEDs are placed annually, and this number is expected to increase with an aging population [2,3,4]. CIEDs are essential components in the treatment of patients with heart failure, malignant arrhythmias, bradycardia, conduction deficits, and congenital heart diseases [3,4,5,6,7].

**Figure 1:**
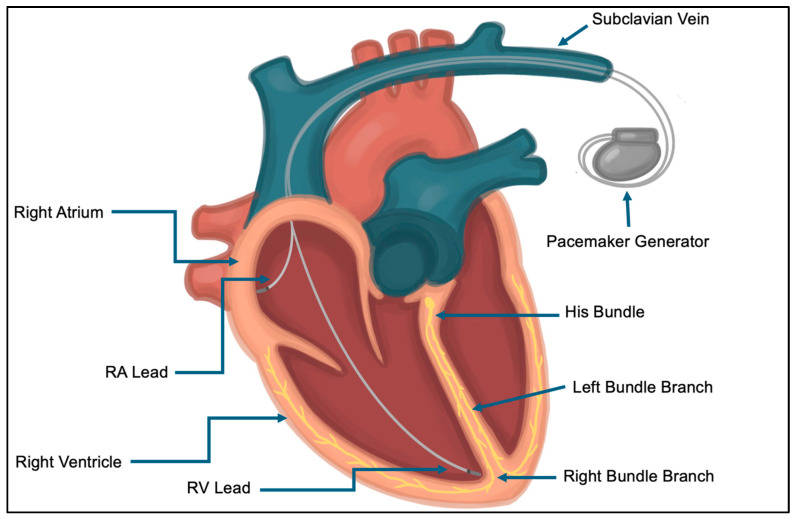
Overview of cardiac anatomy and CIED structure. Example of dual-chamber PPM. RA = right atrium; RV = right ventricle.

**Figure 2:**
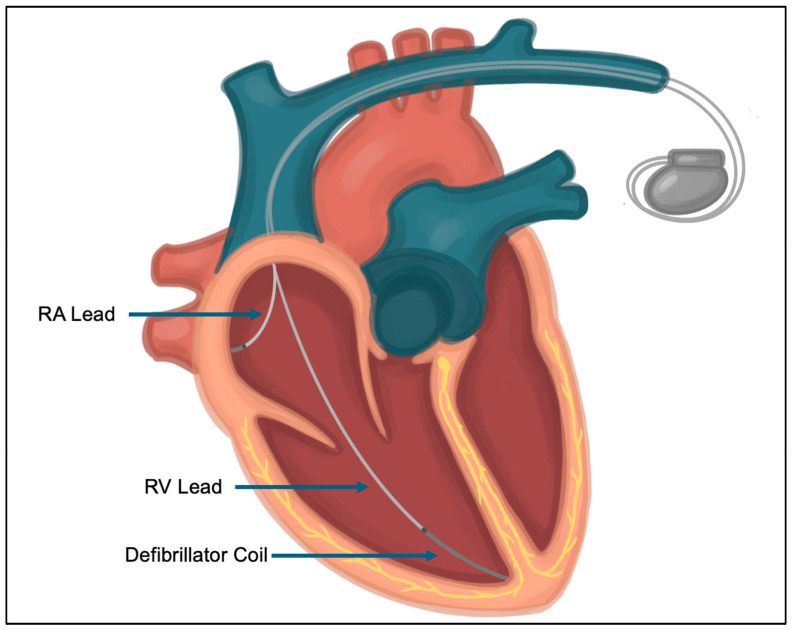
Example of ICD with defibrillator coil along RV lead.

**Figure 3:**
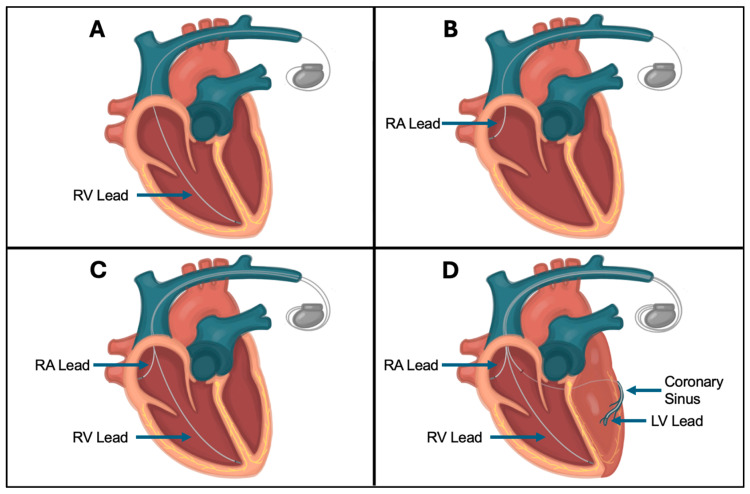
Examples of CIED structure and lead placement; (**A**) single-chamber PPM with RV lead placement; (**B**) single-chamber PPM with RA lead placement; (**C**) dual-chamber PPM with RA and RV lead placement; (**D**) biventricular PPM with LV lead placed in coronary sinus.

Generally, CIEDs comprise two main components—a pulse generator and one or more intracardiac leads (Figure 1). The pulse generator contains a battery, voltage capacitors, rhythm-sensing components, and software. The type and number of leads connected to the generator depends on the indication and type of device. Transvenous pacemaker leads contain electrodes that sense intrinsic cardiac signals and deliver pacing stimulus. Defibrillator leads additionally have one or two coils to allow defibrillation (Figure 2). Transvenous leads may be placed in the right atrium, right ventricle, coronary sinus (epicardial left ventricle), or azygous or subclavian vein depending on a patient’s pacing and defibrillation requirements [8] (Figure 3). Extravascular defibrillators may be placed subcutaneously or within the intrathoracic space in patients who require defibrillation without long-term pacing requirements.

Leadless pacemakers (LL-PPMs) have been more recently introduced to the market: 2016 in the United States [9]. This is largely in response to the limitations of traditional pacing systems, such as infection or valvular impingement. Leadless systems have been shown to provide effective pacing options while avoiding long-term lead-related complications [10,11,12,13,14,15,16,17,18].

With advances in technology surrounding these devices and expanding indications, CIEDs can remain implanted in patients for long periods of time. While implantation procedures have become safer over the years, both the incidence and prevalence of long-term complications are expected to numerically increase. Thus, it becomes increasingly important for providers to be aware of the potential complications of CIEDs in their clinical practice. The purpose of this review article is to review the long-term complications of CIEDs.

## 2. Lead-Related Complications

### 2.1. Lead Failure

Lead failure is a well-established, late-term complication of CIEDs, with several studies demonstrating varying rates of failure depending on lead type and design/structure, implantation technique, and study design [19,20,21,22,23,24,25,26]. While definitions of lead failure may differ, it generally encompasses lead or lead-tip fracture, lead dislodgment, failure to capture or sense, abnormal pacing, and/or defibrillator impedance, extracardiac stimulation, and cardiac perforation. Lead failure can occur due to the dysfunction of any lead component, and risk of failure rates increase over time, varying between 2% and 5% at one year, 2% and 9% at two years, 2% and 15% at five years, and 28% and 40% at eight years [19,20,21,24,27,28,29,30,31,32,33]. These numbers may be skewed, however, as many of these studies included recalled leads with high failure rates or notable safety communications. Through early identification and appropriate management, patients with lead-related complications have similar long-term mortality when compared to matched cohorts [23,25,34,35,36,37].

Several mechanisms of lead failure have been reported, with the most common etiologies including lead fracture, lead insulation damage or breakdown, pace-sensing or high-voltage conductor defects, and artifact oversensing [19,24,25,29,30,31]. Other mechanisms include mechanical damage to the leads from subclavian crush syndrome (the impingement of leads between the clavicle and first rib) and fixation mechanism failure [25,30]. Many risk factors for lead failure have been identified, though it remains difficult to decipher the degree to which mechanical, operative, and patient-specific risk factors contribute to lead failure rates (Table 1). Younger age at implantation, female gender, prior lead failure, lower baseline ejection fraction, and higher baseline functional status are associated with a higher risk of lead failure [28,38,39,40,41,42]. Other patient factors such as congenital heart disease, lower BMI, non-ischemic cardiomyopathy, and a lack of comorbidities have been implicated to increase lead failure risk [28,34,38]. Device-specific characteristics and operative techniques such as a smaller lead diameter, single- versus dual-shock coils, single- versus multi-lumen designs, true bipolar or integrated bipolar leads, insulation material, pectoral versus abdominal implantation, and subclavian versus other access sites have mixed data in several studies [19,20,27,40,41,42,43].

The type of lead used may also influence failure rate. Due to the presence of additional components, defibrillator leads are more likely than pacing leads to fail. A prior meta-analysis demonstrated a 20-fold higher annual failure rate of ICD versus PPM leads [44]. Various models have proven more prone to failure than others, with Sprint Fidelis (Medtronic, Minneapolis, MN, USA), Riata and Riata ST (St. Jude Medical, Sylmar, CA, USA), and Isoline leads (SORIN CRM SAS, Clamart, France) all being recalled due to high fracture rates [45,46,47,48]. However, a recent study on the failure rates of modern ICD leads (Sprint Quattro [Medtronic], Endotak [Boston Scientific, Marlborough, MA, USA], Durata [Abbott, Abbott Park, IL, USA] and Riata ST Optim [Abbott]) showed advancement in lead integrity with a failure rate of only 0.28% per year at median follow-up of 3.2 years with no significant differences between lead brands [49].

The detection of lead failure may manifest as a dysfunction of the CIED, discovered during routine device interrogation, or via alerts from remote monitoring systems. Conductor fracture commonly manifests as high pacing impedance (high electrical resistance where the lead delivers impulses to the myocardium) and rarely loss of capture (myocardium does not respond to electrical stimuli from the device). The fracture of the high-voltage (HV) component can manifest as increases in HV impedance and, rarely, as failure to deliver high-voltage shock. An HV component insulation breach may manifest as low HV impedance and failure to defibrillate. Finally, a pace-sensing lead component insulation breach can manifest as low impedance, noise in the sensing channel with under-pacing (pace-sensing lead), and/or inappropriate shocks.

Lead failure is usually confirmed when one of the following is present on device interrogation: (1) a sudden rise in pacing impedance (>50% rise in 1 week) or fluctuation (≥500 ohms); (2) failure to sense or capture not due to lead dislodgement; (3) ≥2 non-sustained tachycardia events with average R-R cycle length <220 milliseconds not consistent with atrial or ventricular fibrillation; (4) inappropriate shock secondary to sensing of electric noise artifacts from make-break potentials; and (5) sudden rise in defibrillation impedance (>50% rise in 1 week) [42]. Most modern CIEDs are equipped with remote monitoring systems that attempt to detect early lead failure and are important in those with at-risk or recalled leads [40,50]. Additionally, most CIEDs run diagnostic software, such as the Medtronic Lead Integrity Alert (LIA) algorithm, or incorporate such software into remote monitoring systems, such as the Boston Scientific Latitude Lead Alert. The aim of both algorithms is to monitor impedance trends and rapid oversensing to identify lead dysfunction and alert both patients and clinicians before complications arise [51,52,53]. Similar programs, such as the Medtronic Lead Noise Algorithm (LNA) and Abbott Secure-Sense algorithm, have been developed to prevent inappropriate shock therapy in cases of oversensing pace-sensing signals [51,54,55].

When lead failure has been detected, management depends on (1) the etiology of the failure; (2) whether the failure can be corrected without need for lead replacement or revision; or (3) if revision or replacement is needed while weighing the risks and benefits of lead extraction versus abandonment versus conservative management (i.e., specific reprogramming of the device) [19,27,40,52]. Prior studies have shown no differences in outcomes in patients who underwent the replacement of a pace-sensing or ICD lead, though rates of recurrent lead failure are high (16–40% at 5-year follow-up) [19,56]. According to the 2023 HRS consensus statement on remote interrogation and monitoring, those with functioning but recalled leads (e.g., Sprint Fidelis or Riata) need closer follow-up for device interrogation [57].

**Table 1:** Risk factors associated with lead failure.

**Risk Factor**	**Univariate HR (95% CI)**	***p*-Value**	**Multivariate HR (95% CI)**	***p*-Value**	**Paper**
Age (years)					
≥65	0.75 (0.66–0.85)	<0.001			Koneru et al. 2018 [28]
<60	2.01 (1.15–3.53)	0.012	2.33 (1.28–4.24)	0.006	Rordorf et al. 2013 [41]
Gender					
Female	1.56 (1.18–2.08)	0.002			Birnie et al. 2012 [40]
Male	0.80 (0.71–0.90)	<0.001			Koneru et al. 2018 [28]
	0.80 (0.39–1.67)	0.56			Koike et al. 2023 [34]
	0.68 (0.36–1.29)	0.26	0.71 (0.37–1.38)	0.32	Rordorf et al. 2013 [41]
BMI					
	1.01 (0.92–1.10)	0.79			Koike et al. 2023 [34]
	0.99 (0.97–1.02)	0.529			Birnie et al. 2012 [40]
Cardiomyopathy class					
Ischemic	1.68 (0.75–3.76)	0.2			Koike et al. 2023 [34]
	1.16 (0.67–2.00)	0.607	1.97 (1.09–3.55)	0.024	Rordorf et al. 2013 [41]
HCM	1.08 (0.84–1.39)	0.574			Koneru et al. 2018 [28]
	0.29 (0.07–1.20)	0.09			Koike et al. 2023 [34]
	2.19 (1.03–4.64)	0.041			Birnie et al. 2012 [40]
Dilated	0.63 (0.26–1.53)	0.31			Koike et al. 2023 [34]
ARVC	0.91 (0.12–6.64)	0.91			Koike et al. 2023 [34]
Congenital heart disease	3.16 (1.37–7.32)	0.007	2.51 (1.08–5.83)	0.03	Koike et al. 2023 [34]
Other (ARVC, PED, IVF, Congenital)	1.52 (0.93–2.49)	0.095			Birnie et al. 2012 [40]
History of MI	0.84 (0.75–0.95)	0.004			Koneru et al. 2018 [28]
Total number of leads at implantation	0.56 (0.32–0.95)	0.04	0.59 (0.32–1.02)	0.07	Koike et al. 2023 [34]
Previous lead fracture or failure	3.30 (2.14–5.08)	<0.001			Birnie et al. 2012 [40]

Abbreviations: BMI (body mass index); HCM (hypertrophic cardiomyopathy); ARVC (arrhythmogenic right ventricular cardiomyopathy); PED (primary electrical disease); IVF (idiopathic ventricular fibrillation); MI (myocardial infarction). Disclaimer: *p*-values not provided in corresponding papers were calculated based on methods described by Altman and Bland, 2011 [58].

### 2.2. Venous Occlusion

Lead-related venous obstruction (LRVO) is another late-term complication from CIED implantation. LRVO most commonly occurs in the SVC, brachiocephalic, or subclavian veins due to the implantation of transvenous leads into the cephalic or axillary veins. Clinical presentation varies depending on the location, extent, and acuity of the obstruction, as well as the development of collateral venous flow. Most patients are asymptomatic, though those who develop symptoms may experience ipsilateral limb or facial swelling, pain, ecchymoses, or SVC syndrome (facial and neck edema, distended neck and chest veins, headache, blurry vision, or laryngopharyngeal edema resulting in dyspnea, and/or hoarseness).

Symptoms may occur in an “acute” phase within days to weeks, “subacute” phase within the first few months, and “late” phase within months to years [59]. The prevalence of asymptomatic LRVO in patients with CIEDs has been reported at 10–30%, though this may underestimate the true burden [59,60,61,62,63,64]. The incidence of symptomatic LRVO is reported at 0.5–0.9% in the subacute period; however, rates of 5% have been reported in other studies in the late period [60,65]. Superior vena cava (SVC) syndrome due to CIED leads occurs in 1% of patients, but accounts for 30% of total cases of SVC syndrome [66,67].

Multiple risk factors for LRVO have been identified. The strongest predictor of LRVO is the use of multiple CIED leads [62,63,64,65,68]. Other risk factors that have been associated with higher risk for LRVO include reduced left ventricular function, previous transvenous temporary pacemaker leads, malignancy, chronic kidney disease (CKD), history of pocket or endovascular infection, and the duration of lead implantation [59,64,65,68,69]. Several modifiable and non-modifiable factors have been inconsistently identified as risk factors across multiple studies, including patient age, atrial fibrillation, the absence of anticoagulation, and the use of hormonal agents [59,61,63,64,65,68].

**Figure 4:**
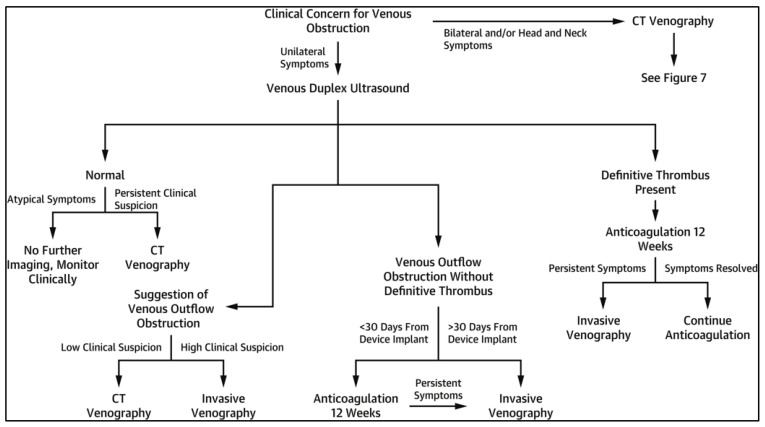
Algorithmic approach for diagnostic evaluation of lead-associated venous occlusion (reproduced with permission from Zimetbaum et al., J. Am. Coll. Cardiol.; published by Elsevier, 2022) [59].

Diagnostic evaluation of suspected LRVO varies based on patient symptoms, comorbidities, and the suspected vein occluded. Venous duplex ultrasound (VUS) is a common initial diagnostic test, as it is quick and inexpensive and spares patients from radiation exposure. Diagnostic utility may be limited, however, by factors such as patient habitus, operator experience, lead artifacts interfering with color Doppler imaging, and the ability to compress deep vessels such as the subclavian or SVC [59,70]. Previous studies have demonstrated that 21% of patients with proven venous thoracic outlet syndrome had false-negative VUS [71]. Cross-sectional imaging with CT/CT venography or MR/MR venography can thus be used in patients with normal VUS in which high clinical suspicion remains, or in those with indeterminate studies. While these studies may also be limited by metallic artifacts, previously described protocols help minimize this well-known problem [72,73]. Invasive studies such as intravascular ultrasound (IVUS) may also be used for further characterization, though invasive catheter venography remains the gold standard for diagnosis and offers benefits in situations where endovascular intervention is planned [70]. Zimetbaum et al. suggested a diagnostic flowchart for the evaluation of suspected LVRO based on patient symptoms, degree of clinical suspicion, suspected location of the obstruction, and timing from device implant (Figure 4) [59].

Management depends on patient symptoms and the location of the occlusion [59,74]. Typically, cases of asymptomatic LRVO do not require specific treatment, and most patients with symptomatic LRVO receive conservative or no treatment [65]. For patients in whom the placement of a new lead through the obstruction is planned, contralateral tunneled lead implantation, redundant lead extraction, or revascularization procedures can be considered on an individualized basis with shared decision making [51]. A trial with a direct oral anticoagulant for 3–6 months in those with distal symptomatic LRVO can also be considered as initial management for appropriate candidates [59].

Proximal symptomatic occlusions in the brachiocephalic vein or SVC, which occur in approximately 15% of patients based on large-database studies of Medicare and Medicaid patients, may require invasive interventions [65]. The current Heart Rhythm Expert Consensus Statement on CIED lead management and extraction recommends the removal of involved leads, which has been shown to decrease LRVO-related health care utilization by 40% [51,65]. Lead extraction has been shown to be the most commonly pursued treatment in these cases (74% of patients with invasive intervention), followed by revascularization procedures (26%) such as balloon venoplasty, thrombolysis, stenting, and thrombectomy [65]. While the extraction of involved leads may facilitate further revascularization techniques, Ferro et al. showed that only a small proportion of patients undergo both extraction and percutaneous revascularization (1.1% of those with initial extraction and 3.3% of those with initial revascularization) [65].

Chronic LRVO is due to a combination of factors including inflammatory and fibrotic changes in the vascular endothelium as well as thrombus formation. The primary goals in these cases are to (1) restore patency through the removal of leads, (2) dilate the lesion through angioplasty if anticoagulation is unsuccessful, or (3) carry out stenting for those with angioplasty complications or recalcitrant stenosis [75]. These endovascular interventions carry the risk of lead dysfunction, dislodgement, or infection, and stent placement over existing leads with the subsequent “jailing” of the lead has been previously reported with concerns for future difficulty with lead removal [75]. Surgical management, such as saphenous or femoral venous grafting is typically reserved for those in whom endovascular interventions are not feasible or have failed.

Many studies have assessed long-term patency rates for those with lead-related central venous stenosis or obstruction (CVS/CVO) undergoing invasive management. Asif et al.’s retrospective study on 28 hemodialysis patients with lead-related CVS who underwent balloon angioplasty showed clinical success in 27/28 patients (96%) with patency rates of 18% at 6 months and 9% at 12 months prior to angioplasty, and secondary patency rates of 95% and 86% at 6 and 12 months, respectively, after the procedure [76]. Saad et al. analyzed 14 patients with stent placement for CVS/CVO in the setting of ipsilateral hemodialysis arteriovenous access and noted no instances of CIED or lead failure and demonstrated patency rates of 45% at 6 months and 9% at 12 months [77]. Borsato et al.’s retrospective study of 13 patients with clinically significant CVS or CVO with indwelling PM leads who received balloon angioplasty and stenting demonstrated patency rates of 87.5% at 30 days, 75% at 60 days, and 50% at 120 days [75]. In these patients, there was no evidence of PPM or lead dysfunction, infection, or revision at 30 or 90 days [75].

### 2.3. Tricuspid Regurgitation

Right ventricular lead placement may result in valvulopathy by impinging upon the tricuspid valve leaflets or subvalvular apparatus, or through the development of lead-related dyssynchrony [78,79,80,81,82,83,84,85]. The frequency of significant tricuspid regurgitation (TR) (defined as ≥2+) after undergoing CIED implantation varies widely and has been reported to occur in 7–45% of patients [78,79,80,81,82,84,85,86,87,88,89,90,91,92,93,94]. Tricuspid valve dysfunction following CIED implantation may be asymptomatic but may result in right-sided heart failure. Prior studies have shown higher rates of significant TR in patients with CIEDs, and outcomes from these studies have shown associations between device-related TR and increased heart failure hospitalization, morbidity, and mortality when compared to risk-matched cohorts [80,84,86,87,95].

Risk factors for the development of significant TR after CIED implantation have not been well evaluated and most data are observational. Patient-specific risk factors include advanced age (>70 years old), diabetes mellitus, chronic obstructive pulmonary disease (COPD), persistent atrial fibrillation, and peripheral arterial disease [82,87,90,96]. In a study on 382 patients with either a PPM or ICD with an available pre- and post-device TTE, Lee et al. found that an increased RA area (25.4 ± 8.0 cm^2^ (*p* = 0.001)), elevated RVSP (46.4 ± 11.5 mmHg (*p* = 0.001)), increased LA area (27.6 ± 6.3 cm^2^ (*p* = 0.003)), and pre-existing moderate or greater MR (*p* = 0.001) were clinically significant risk factors for the 38 patients with increased TR by ≥2+ grades [90]. Other previously characterized echocardiographic findings include pre-existing mild TR, moderate to severe aortic insufficiency (AI), and elevated RA-RV pressure gradient [96]. These data taken together suggest that predisposition to TR (e.g., existing valvular heart disease, evidence of right-sided dysfunction, increased pulmonary artery pressures) may be unmasked after CIED placement, but determining causality is difficult.

Certain device-related risk factors have also been identified with mixed data. The number of leads crossing the tricuspid annulus in some studies have demonstrated an increased risk [97,98] and in others no differences [90,99]. Some studies have shown an increased risk of TR when leads are placed between the septal and posterior leaflets [91]; however, other studies have not shown such an association [100]. Thicker/stiffer ICD leads have also been suggested to increase the risk for worsening TR [81,82,84,85,87,91].

The diagnosis of CIED-related tricuspid valve dysfunction is made primarily through transthoracic or transesophageal 2D or 3D echocardiogram with Doppler imaging. However, an assessment of the tricuspid apparatus with 2D echocardiography, both with a transthoracic and TEE approach, is often limited in that in any given view, only two of the leaflets are readily visualized, and the sensitivity of 2D TTE in detecting CIED lead-related TV dysfunction has been reported between 12 and 17% [79,83,87,91,101,102]. In comparison, 3D TTE/TEE has improved diagnostic utility in detecting CIED-related TR, with sensitivity approaching 94% in some studies [91]. This modality allows for the simultaneous visualization of all TV leaflets as well as the location of the device lead with relation to TA [101,103,104,105,106].

Clinical factors that guide management include evidence of symptomatic right heart failure, severity of TR, extent of lead-related valvular damage, the degree of RV dysfunction, and tricuspid annular dilation [79]. Management strategies include medical therapy, transvenous lead extraction (TLE), and percutaneous and surgical interventions. Conservative therapy is usually reserved for those with mild or moderate TR, or patients who are poor surgical candidates with severe RV dysfunction or pulmonary hypertension. There are limited long-term outcome data on conservative medical therapy for lead-related TR [87]. Regarding lead extraction, there is currently no consensus recommendation in the 2017 HRS guidelines for lead extraction in the setting of lead-related TR (in the absence of infection) [51]. The decision to pursue lead extraction in the context of suspected lead-related TR involves weighing risks of lead-removal complications (such as worsening TR or further damage to the tricuspid apparatus, reported between 0.4 and 5.6%) [79,87] versus the increased morbidity and mortality risk of untreated lead-related severe TR [80,86]. Thus, when the operative risk is low, lead extraction should be considered in applicable candidates [79]. There have been conflicting data on this subject, with some studies demonstrating 63% improvement in TR severity after lead extraction for symptomatic TR [107], whereas other studies have shown no improvement [108].

Surgical repair for lead-related severe TR includes suture or ring annuloplasty, and TV repair/replacement with or without lead retention. In patients with severe primary iatrogenic TR (including lead-related TR), surgical intervention can be considered for those who remain symptomatic or with progressive RV dilation/dysfunction despite maximally tolerated medical therapy or non-surgical lead intervention [109]. Extraction with reimplantation sparing the tricuspid valve (such as through the placement of an epicardial lead or leadless pacemaker) may be considered [79]. Both the 2020 AHA/ACC and 2021 ESC/EATCS guidelines for the management of valvular heart disease prefer valve repair over replacement, but replacement may be necessary in cases of marked annular dilation or dysfunctional/damaged leaflets [109,110].

### 2.4. CIED Lead Placement and Fixation Techniques

Various techniques for lead placement and fixation have been described, as well as the type of technique used for broader implications on lead-related complications. Venous access is essential for CIED lead placement, and the exact approach depends on operator experience and preference [111,112]. The most commonly used veins for lead placement are in the pre-pectoral area of the upper chest, including the subclavian, axillary, and cephalic veins [111]. Historically, subclavian vein puncture (SVP) has been the most common approach to venous access for CIED leads [113]. However, several studies have shown increased rates of periprocedural complications related to SVP, including pneumothorax and crush syndrome (a condition in which a lead is crushed between the clavicle and first rib, resulting in lead failure) [114,115]. The incidence of pneumothorax after lead placement using SVP is estimated between 1.3 and 2.4% [116,117]. Because of this, approaches such as axillary vein puncture (AVP) and cephalic vein cutdown (CVC) have been explored. A large metanalysis of 36 observational studies and RCTs showed that both CVC and AVP had reduced odds of pneumothorax (OR: 0.193 and 0.128, respectively) and lead failure (OR: 0.63 and 0.41, respectively) compared to SVP [118]. A separate metanalysis of >35,000 patients across 23 studies similarly showed that SVP was associated with higher risk of pneumothorax compared to CVC (RR, 4.88; *p* < 0.05) [115]. This study also showed no differences in periprocedural complications between the CVC and AVP approaches; however, the AVP approach was associated with significantly higher procedural success than the CVC approach (96.3% vs. 76.2%; *p* < 0.05) [115]. These findings were subsequently affirmed in the ACCESS trial, which showed that intra-pocket ultrasound-guided AVP was superior to the CVC approach in terms of procedural success (99% vs. 86.9%, respectively; *p* < 0.05), time to venous access, procedure duration, and radiation exposure without any differences in periprocedural complications [119]. Overall, these data suggest that both CVC and AVP are safe and effective for CIED lead placement when compared to SVP, and AVP has additional advantages given its reduced procedural time and improved procedural success compared to other methods.

There are two primary methods of lead fixation: active fixation (which utilizes a screw-in helix to secure leads to the myocardium) and passive fixation (which utilizes tines or fans to passively lodge leads in trabeculae) [112]. Regarding complication rates, actively fixated leads generally have a more favorable safety profile compared to passively fixated leads, including lower rates of lead dislodgement [120], lead infection [121], and lower pacing thresholds [122]. These benefits have been observed for both atrial leads [123] and ventricular leads [124]. This difference in safety outcomes is largely due to improved lead stability and placement precision with active leads. However, active fixation is associated with certain disadvantages, including longer procedure and fluoroscopy times [120], more challenging lead extraction [125], and increased risk of cardiac perforation [126,127]. When deciding on the appropriate fixation technique, factors such as advanced age (>80 years) and female sex should be considered, as both are associated with increased risk of cardiac perforation [127].

### 2.5. Leadless Pacemakers and Emerging Technologies

First implemented in 2012 [10], leadless pacemakers (LPMs) have demonstrated significant advantages over traditional CIEDs. These devices are small (~42 mm × 6 mm) and actively fixated to the RV apex, features that have shown to reduce rates of infection or dislodgement. A prospective study on ~6000 patients showed that patients with a Micra LPM versus transvenous single-chamber PPM had significantly lower rates of device-related infection (<0.2% vs. 0.7% HR 0.68; *p* < 0.01), dislodgement (0.4% vs. 1.3%; *p* < 0.01), device re-intervention (3.6% vs. 6%; *p* < 0.01), and heart failure hospitalization (19.9% vs. 22%; *p* < 0.01) at 3-year follow-up [128]. Other studies have shown similar results regarding the long-term complication of LPMs, including reduced rates of tricuspid regurgitation [129,130,131]. However, LPMs have been associated with higher rates of periprocedural and in-hospital complications. A large retrospective study of approximately 27,600 patients from the NIS database found that those receiving LPMs had significantly higher odds of in-hospital mortality (OR, 1.63; *p* < 0.001), vascular complications (OR, 7.54; *p* < 0.001), venous thromboembolism (OR, 3.67; *p* < 0.001), and device thrombus (OR, 1.54; *p* < 0.001) compared to those receiving transvenous single-chamber PPMs [132]. Other studies have also shown higher rates of periprocedural pericardial effusion, pericarditis, and cardiac perforation in LPM versus transvenous PPMs [129,130]. Risk factors associated with periprocedural complications of LPM placement include end-stage renal disease, congestive heart failure, and coagulopathy [133]. These data suggest that, although LPMs have higher rates of periprocedural complications, their long-term safety profile offers advantages over transvenous CIEDs and their performance is likely to improve with further research [134]. Other emerging technologies that are worth mentioning include small-diameter lumenless CIED leads [135] and bioresorbable CIED leads for temporary cardiac pacing [136]; however, these devices are still in development, and data on patient or clinical outcomes are limited.

## 3. CIED-Related Arrhythmias

### 3.1. Pacemaker-Induced Cardiomyopathy

**Figure 5:**
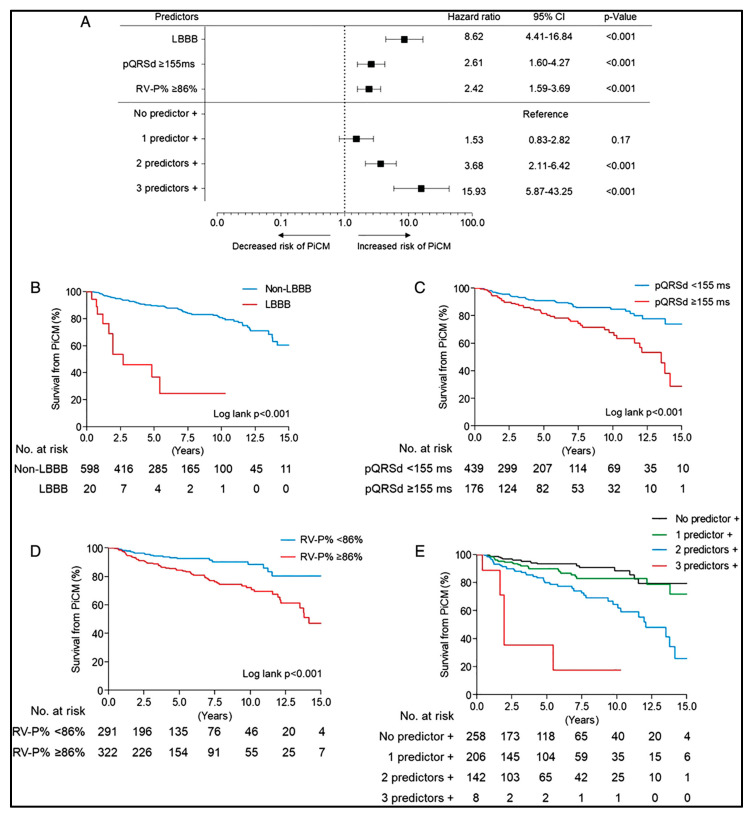
Hazard ratios of pacing-induced cardiomyopathy (PiCM) and PiCM survival curves based on predictors; (**A**) forest plot of hazard ratios of PiCM predictors; (**B**–**E**) Kaplan–Meier curves of individual and combined predictors, including left bundle branch block (LBBB), paced QRS duration (pQRSd) ≥ 155 ms, and right ventricular pacing percentage (RV-P%) ≥ 86% (reproduced with permission from Cho et al., Eur. J. Heart Fail.; published by Wiley, 2019) [96].

Pacemaker-induced cardiomyopathy (PiCM) is defined as left ventricular systolic dysfunction resulting from frequent right ventricular pacing. PiCM is generally defined as a decrease in the LV ejection fraction by ≥10% with a final LVEF ≤ 50% or a newly reduced LVEF ≤ 40% after the initiation of ventricular pacing [137,138,139]. These changes can be seen within the first months after the initiation of pacing, and the risk increases over time (Figure 5) [96,137,138,139]. The suspected mechanism for this is ventricular desynchrony (typically in the context of right ventricular pacing) leading to the delayed activation of the basolateral LV, which then redistributes myocardial strain (i.e., myocardium closest to pacing experiences early systolic shortening and impaired contractile function), resulting in inefficient myocardial work and altered myocardial metabolism [137,140]. This ultimately leads to increased cardiac filling pressures and reduced cardiac output [141]. The incidence of this complication varies by study but has been shown to occur in 10–20% of pacemaker recipients with predominantly RV pacing within 1 year after pacer implantation, followed by gradual decrement over additional years [138,139,142].

A large metanalysis identified several possible risk factors for the development of PiCM, including male sex, baseline QRS duration ≥ 120 ms paced QRS duration ≥120 ms, increased percentage of RV pacing, history of MI, or history of atrial fibrillation (Table 2) [142]. Both the RV pacing burden and paced QRS duration have shown a proportional relationship with PiCM risk [142]. Prognostically, PiCM confers significant morbidity and mortality. In a retrospective study of 618 patients who underwent pacemaker placement, patients who developed PiCM (14.1%) had higher rates of cardiac death (HR, 5.7; CI, 1.19–21.8), heart failure hospitalization (HR, 5.11), and combined risk of heart failure hospitalization or all-cause death (HR, 2.93) compared to patients who did not develop PiCM (see Figure 5) [96]. Additionally, mortality was increased in patients who developed PiCM who also had a combination of baseline LBBB, increased RV pacing (≥86%), and increased paced QRS duration (≥155 ms) [96]. Similar outcomes were also observed in an analysis of the DAVID trial, which found that a high burden of RV pacing (≥40%) resulted in increased heart failure hospitalization at 18 months compared to a low burden of RV pacing (≤10%) in patients with baseline reduced systolic function (LVEF ≤ 40%) [143]. These findings suggest that an increased burden of RV pacing increases the risk of PiCM and may worsen systolic function in patients with established cardiomyopathy, both of which confer poor prognosis in terms of mortality and CHF hospitalization.

**Figure 6:**
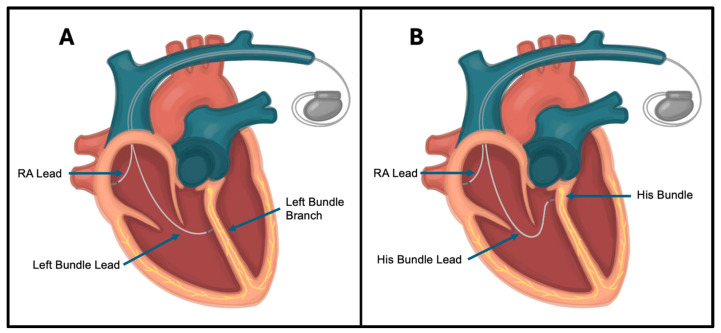
Examples of CSP: (**A**) left bundle branch pacing (LBBP) with RV lead located along intraventricular septum near left bundle branch; (**B**) His bundle pacing with RV lead located along intraventricular septum near His bundle.

The management of PiCM usually involves device upgrades to CRT or conduction system pacing (CSP), which is a method of pacing that simulates the heart’s native conduction system. CRT is accomplished by adding another lead for LV pacing (routed through the coronary sinus) in addition to the lead providing RV pacing. A large metanalysis found that CRT upgrade in patients with PiCM improved LVEF by 11–19% within 6 months of device upgrade [142]. This same analysis showed a similar benefit with His bundle pacing [142], which is accomplished by directly pacing the bundle of His with the RV lead (Figure 6). Of note, no studies in this analysis demonstrated benefits in terms of mortality but did show improved LVEF, CHF symptom burden, and reduced CHF hospitalization in PiCM patients who underwent CRT upgrade [142]. Similar results were shown in the PACE trial, in which 177 patients with normal LVEF (average, 62.2%) who underwent pacemaker placement were randomized to RV pacing or CRT in a double-blind fashion. Twelve-month follow-up showed that patients in the CRT arm had preserved LV systolic function (mean LVEF, 63.3%) while patients in the RV pacing arm had reduced function (mean LVEF, 56.2%), which continued to worsen at four-year follow-up (mean LVEF, 53.2%) [144]. The CRT arm also had reduced rates of CHF hospitalization compared to the RV pacing arm (23.9% vs. 14.6%, respectively) [144]. These data support that device upgrade from single-ventricle pacing to either CRT or His bundle pacing improves LV function and CHF symptoms and lowers CHF hospitalization rates in patients with PiCM.

More recent studies have evaluated CSP with left bundle branch pacing (LBBP) for both the prevention and treatment of PiCM. LBBP is defined as the capture of left bundle (either the main trunk or one of its proximal fascicles) along with septal myocardium at low output [145] (Figure 6). As described above, His bundle pacing is another technique sometimes used to help improve mechanical synchrony utilizing the native conduction system, and many studies have demonstrated its clinical benefits and feasibility [146,147,148,149,150]. His pacing, however, is often limited by difficulty with implantation, higher pacing thresholds, lower R-wave amplitudes, lower success rates in those with prolonged QRS, the development of distal conduction block, and early battery depletion [147,150,151,152,153,154,155]. An His-SYNC study showed a failure of QRS normalization with HBP pacing in about 50% of patients with underlying LBBB [156]. Thus, LBBP was developed to overcome these limitations. Advantages of LBBP compared to His bundle pacing include a wider target area for lead placement (making implantation easier to perform with less precision) [157,158], the ability to pace more distally in the conduction system (effectively bypassing pathological areas) [157,159,160], and a narrow-paced QRS (typically <130 ms), which provides a lower and more stable capture threshold (improving device longevity) [157,158,161,162].

When compared to RV pacing, LBBP may have significant advantages. A 2022 multicenter observational study of 703 patients undergoing PPM placement found that patients with LBBP had significant reductions in a composite outcome of mortality, heart failure hospitalization, or upgrade to biventricular pacing compared to patients with RV pacing (10% vs. 23.3%; *p* < 0.001) [163]. The improved outcomes with LBBP versus RV pacing may be attributed in part to a reduced incidence of PiCM with LBBP. For example, one prospective, single-center study of 151 patients undergoing PPM placement found that LBBP did not result in significant reductions in LVEF at 23-month follow-up [164]. In fact, some evidence suggests that LBBP can help reverse the changes in PiCM due to RV pacing. A small prospective study of 20 patients with PiCM related to RV pacing (>70%) showed that transition to LBBP led to significant increases in LVEF (32% to 47%; *p* < 0.001) and significant reductions in QRS duration (193 ± 18 ms to 130 ± 17 ms; *p* < 0.001) at 6-month follow-up [165].

LBBP achieved widespread use in 2017, and more studies are needed to assess long-term complications of this technique. Known complications include septal perforations, lead dislodgements, lead fracture, right bundle branch (RBB) injury, thrombosis, and septal artery insult [161,166,167,168,169,170,171,172,173,174,175,176,177]. Lead dislodgement has been shown to occur in 0.3–3.1% of patients undergoing LBBP [167,169,178,179,180]. Efforts to reduce the risk of dislodgement should be aimed at ensuring adequate slack in the leads, appropriate pacing parameters, and repositioning the lead at a different site in cases of perforation. Perforation into the LV is a common complication, having been reported to occur in between 0.3 and 6.0% of patients [166,167,169,178,179,180,181]. This is due to the need for the deep implantation of the leads into the subendomyocardium of the LV. Delayed septal perforation has been reported between 0.08 and 0.33% of cases [166,169,178]. Intraprocedural RBB injury is generally transient and recovers before the end of the procedure [176], but may lead to complete block in those with a pre-existing LBBB.

Compared to RV pacing, however, LBBP appears to have similar rates of procedure-related complications. A large, prospective, multicenter trial by Palmisano et al. studied the long-term risk of device-related complications [182]. The study included 1029 consecutive patients receiving RV pacing, LBBP, or His bundle pacing and compared these groups using propensity score matching. Overall, they showed no significant differences in device-related complications between patients undergoing LBBP versus right bundle pacing (RBP) (1.3% vs. 3.5%; *p* = 0.358). These studies suggest that LBBP could be an effective alternative to RV pacing and may be useful in both the treatment and prevention of PiCM. More long-term studies will be valuable in assessing LBBP outcomes and associated complications.

### 3.2. Pacemaker Syndrome (Loss of AV Synchrony)

Pacemaker syndrome is defined as the intolerance of ventricular-based pacing due to loss of synchrony between atrial contraction, atrioventricular valve opening/closing, and ventricular contraction [183]. This is most often diagnosed clinically based on a spectrum of symptoms related to hemodynamic and rhythm abnormalities, including fatigue, presyncope/syncope, chest discomfort, dyspnea, and new onset/worsening heart failure (e.g., jugular venous distention, edema, orthopnea, exertional dyspnea) [184]. The incidence of pacemaker syndrome varies based on study criteria but occurs most commonly in single-chamber pacers set in the VVI mode [184]. Two large multicenter trials showed that the incidence of pacemaker syndrome-related symptoms occurred in 16% of patients by 12 months [183] and 26% of patients by 18 months [185]. Of note, no baseline risk factors (besides device type and settings) have been correlated with pacemaker syndrome development [186]. As described previously, prolonged RV pacing can also cause new onset or worsening heart failure [137,140,142], so the diagnosis of pacemaker syndrome can be challenging and is often one of exclusion.

When pacemaker syndrome develops, management often involves device upgrade from single- to dual-chamber pacing. Several studies have shown that this upgrade improves quality of life in affected patients and reduces the risk of developing atrial fibrillation [185,187,188,189]. However, no study to date has shown benefits for all-cause mortality, cardiovascular mortality, stroke, or heart failure hospitalization in patients who undergo device upgrade for pacemaker syndrome [188,189]. Additionally, upgrade to dual-chamber pacing carries risk, as these devices are more expensive, more complex to implant, and carry higher risk of perioperative complications (e.g., atrial lead displacement and pneumothorax) [188,189,190].

### 3.3. Inappropriate Device Shocks

Inappropriate shocks are a notable complication of ICDs and carry significant risks to patients. In 1996, a large prospective study of 1544 primary and secondary prevention ICD recipients showed that 18% of patients received at least one inappropriate device shock at 5-year follow up [191]. An analysis of the 1997 MADIT II trial showed that of the 719 enrolled patients who received primary prevention ICDs, 13% experienced an inappropriate shock by 2-year follow-up [192]. With advances in ICD technology, the rate of inappropriate device shock is expected to decrease with time; however, more recent studies still demonstrated a significant risk of inappropriate shock. An analysis of the 2009 EFFORTLESS trial showed that the rates of inappropriate device shocks were 8.7% and 16.9% at one and five years post implant, respectively [193]. Similar results were also seen in the analysis of the 2011 PRAETORIAN trial, which showed a rate of inappropriate shocks of ~10% at one year and ~15% at five years for both subcutaneous and transvenous ICDs [194].

Inappropriate device shocks can occur within months of implantation and cumulative incidence increases over subsequent years [191]. Several mechanistic causes have been described in the literature. The most common cause is inappropriate device oversensing of another tachycardic rhythm, such as atrial fibrillation, atrial flutter, or supraventricular tachycardia [191,192]. Another cause is device malfunction, such as T-wave oversensing or ICD lead dislodgement/failure (a rare cause of inappropriate shock) [21,195,196]. In taking these mechanisms into consideration, the strongest risk factors for inappropriate shocks are a history of atrial fibrillation/atrial flutter or SVT [191,192,197]. Other risk factors include previous cigarette smoking, age < 70 years at device implantation, and late-stage heart failure (NYHA class III or IV) [191,192,197]. Of note, studies have shown no significant difference in the risk of inappropriate shock based on device type (e.g., single-chamber ICD, dual-chamber ICD, or cardiac resynchronization therapy defibrillator (CRT-D)) [191].

Inappropriate device shocks carry significant prognostic importance. Several studies have shown direct correlation between inappropriate shocks and mortality risk [191,192,198]. Mortality risk also increases proportionally to the number of inappropriate shocks [191]. The most common cause of death after inappropriate device shock is progressive heart failure followed by cardiac arrhythmia [191,198]. Of note, appropriate device shocks carry a higher mortality risk than inappropriate device shocks [191,198], which is likely attributable to the underlying malignant cardiac arrhythmia (e.g., ventricular tachycardia (VT) or ventricular fibrillation (VF)), which triggered the shock. However, concomitant inappropriate and appropriate shocks increase the risk of death compared to either on its own [198]. One explanation for this is the harmful mechanical, arrhythmic, or hemodynamic effects from the shock itself [192,196]. This could also be attributed to the underlying comorbidity that triggered the shock (such as atrial fibrillation or acute decompensated heart failure).

Several strategies have been studied to address inappropriate device shocks. A single-center, prospective study of ~1600 patients with ICDs set between 1996 and 2006 showed no difference in the percentage of inappropriate ICD shocks in patients who received an ICD before or after 2004, suggesting that improved ICD technology/software had not reduced inappropriate shock incidence to that date [191]. An important confounding factor to consider when assessing these data, however, is that the indication for ICD placement has shifted over time from predominantly secondary prevention to primary prevention. Patients who underwent primary prevention ICD placement are more likely to have worse cardiac condition (e.g., ischemic heart disease, heart failure, history of heart failure hospitalization) [199,200], which may increase their risk for inappropriate device shocks.

More recent studies have shown promising results with device reprogramming. A 2012 prospective study of 1500 patients with primary prevention ICDs showed that reprogramming therapy thresholds for tachyarrhythmias ≥200 bpm or with prolonged delay for rates 170 bpm were associated with reductions in inappropriate device therapy and all-cause mortality (compared to conventional programming) [201]. These methods for ICD programming are recommended in current guidelines [202]. The impact of different device types (e.g., single-chamber ICD, dual-chamber ICD, CRT-D) on inappropriate shock risk has also been evaluated but have not shown a significant correlation. For example, a large metanalysis showed no differences in inappropriate device shock risk in the recipients of all of these types of cardiac devices [191]. Other studies have also shown no differences in inappropriate shock risk between single-chamber ICD and dual-chamber ICD [203,204]. Some groups have investigated the use of combined electrogram interpretation with physiologic measurements to distinguish VT/VF from other forms of arrhythmia with increased fidelity [205], but no large studies of this method have been published to date. These data taken together suggest that device reprogramming (such as prolonged detection time and high-rate programming) is an effective method for reducing the incidence of inappropriate device shocks [202].

### 3.4. Pacemaker-Mediated Tachycardia and Repetitive Non-Reentrant Ventriculoatrial Synchrony

Repetitive retrograde ventriculoatrial (VA) conduction in patients with dual-chamber pacemakers may cause one of two forms of VA conduction abnormalities: pacemaker-mediated tachycardia (PMT; also referred to as “Endless Loop Tachycardia”) and repetitive non-reentrant ventriculoatrial synchrony (RNRVAS) [206,207]. Both these arrhythmias occur when the atrial lead of the pacer detects a retrograde *p*-wave and are differentiated by device pacing mode and when the P-wave falls within the post-ventricular atrial refractory period (PVARP). PMT may occur when a device is set to P-synchronous ventricular pacing (DDD, DDDR). For PMT to occur, the retrograde conduction of a ventricular beat (e.g., premature ventricular contraction (PVC)) travels through the AV node or an accessory pathway, is sensed by the atrial lead outside of the PVARP, and results in ventricular activation and subsequent additional retrograde conduction, which establishes a re-entrant circuit of tachycardia [206]. In contrast, RNRVAS occurs when a device is set to AV sequential pacing (DDD, DDR, DDI, DDIR) and retrograde conduction falls within the PVARP, which leads to atrial undersensing, the elongation of the atrioventricular (AV) interval, and AV dyssynchrony [207].

Both of these pacemaker-related arrhythmias result in unfavorable hemodynamics with either tachycardia or asynchronous AV conduction in PMT and RNRVAS, respectively. The risk of PMT is increased in devices with a short PVARP interval, whereas RNRVAS is predisposed in devices with a long PVARP interval, long AV delay, and high amount of AV sequential pacing [207]. These arrhythmias can be managed with adjusting device settings. For instance, PMT can be addressed by either increasing the duration of the PVARP or adjusting the atrial lead to only sense sinus P-waves and not retrograde P-waves [206,208]. Of note, many pacemakers have built-in algorithms that help prevent PMT by automatically prolonging the PVARP whenever a retrograde P-wave is sensed [209]. Lastly, PMT can be treated acutely with the placement of a magnet over the pacer to disable pacing functionality [210]. RNRVAS can be prevented by lowering the lower rate limit (LRL) or upper sensor rate, disabling rate-response or rate-drop response, shortening AV delays, shortening the PVARP, or by extending non-competitive atrial pacing to allow atrial recovery and capture. Unlike PMT, there are no built-in device algorithms to address RNRVAS.

## 4. CIED Infections

The exact definition of CIED infection is imprecise given a wide variety of presenting syndromes and the absence of a diagnostic gold standard. The 2017 HRS Consensus Statement on CIED Lead Management and Extraction categorizes CIED infections as either pocket or systemic [51]. Isolated pocket infection is limited to the subcutaneous (or submuscular) location of the generator or leads with no evidence of bacteremia. Systemic CIED infection differs from pocket infection in that these cases involve evidence of vegetations on heart valves or device leads (i.e., CIED-related infective endocarditis), bacteremia, or sepsis. Systemic infections start with bacterial contamination of either the device pocket or leads, which subsequently spread to local structures (e.g., heart valves, other device leads) or in a hematogenous manner. Systemic infection is frequently associated with endocarditis and often interchangeably termed CIED-IE (or device-related infectious endocarditis), with *S. aureus* being the most commonly implicated organism [51]. A retrospective study of CIED-related infections from 1991 to 2003 found that CIED-IE comprised about 10% of cases of device infection [211].

**Figure 7:**
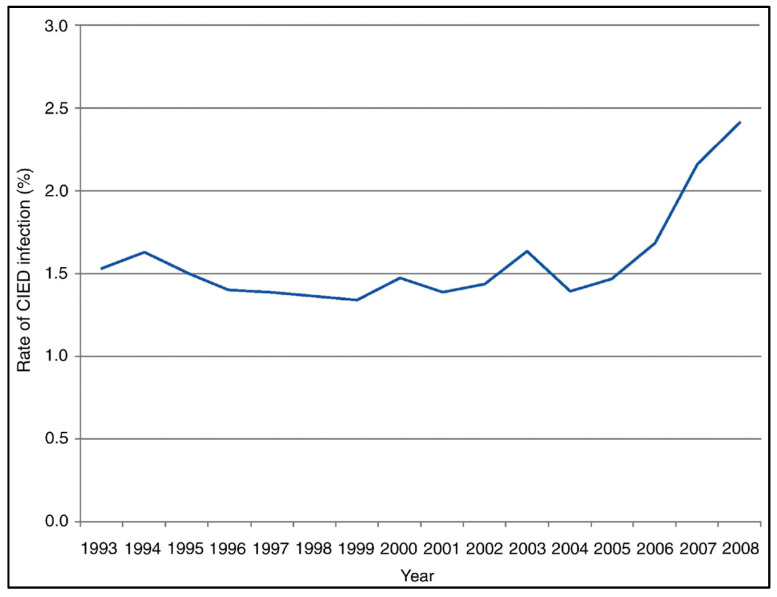
Trends in annual CIED-related infection rate from 1993 to 2008 (reproduced with permission from Greenspon et al., J. Am. Coll. Cardiol.; published by Elsevier, 2011) [212].

As cardiac devices are being implanted more frequently, CIED infection has also become more prevalent. With data drawn from the National Inpatient Sample (NIS) from 1993 to 2008 in the United States, Greenspon et al. found 69,000 cases of device infection among 4.2 million primary CIED implantations, translating to a 1.61% cumulative incidence of device infection over a 16-year period (see Figure 7) [212]. Concurrently, cumulative CIED implantation has increased by 96% and was associated with an increased rate of infection from approximately 1.5% in 1993 to 2.4% in 2008 [184]. Another retrospective population-based study by Uslan et al. found an incidence of 1.9 per 1000 device-years [213]. There also appears to be a correlation between cardiac device type and infectious risk. A retrospective study of ~1500 U.S. patients with CIEDs showed a higher infectious rate with ICDs versus PPMs (8.9 vs. 1.9 per 1000 device-years, respectively) [213]. Another large retrospective study of approximately 98,000 Danish patients undergoing CIED implantation showed similar findings, with the incidence of infection among patients with a PPM, an ICD, cardiac resynchronization therapy with a pacemaker (CRT-P), or CRT-D at 1.2, 1.9, 2.2, and 3.4%, respectively, during the device’s lifetime [214].

Pocket infection is primarily a clinical diagnosis. Patients with these infections typically present with local signs and symptoms of inflammation, such as tenderness, wound dehiscence, erythema, warmth, purulence, and skin breakdown exposing leads or the generator [51]. A CIED that has eroded through the skin and is exposed is presumed to be infected [215]. Disseminated infection typically presents with systemic symptoms (e.g., fever, chills, fatigue) but may also present with signs and symptoms of pocket infection [51]. In cases where there is evidence of pocket infection and the Duke criteria for IE are satisfied, systemic infection is confirmed.

There are multiple patient- and procedure-related factors that increase the risk of infection (Table 3). A 2015 meta-analysis of 180,004 patients showed that patient-specific risk factors included previous device infection, diabetes mellitus (DM), end-stage renal disease (ESRD), COPD, malignancy, heart failure, recent corticosteroid use [216]. Procedure-related risk factors were identified as device pocket hematoma device revision/replacement, reoperation for lead dislodgement, lack of prophylactic antibiotics, and long procedure duration [216]. As mentioned previously, the type of device also impacts infectious risk, with ICDs and CRTs conferring a greater risk of infection compared to PPMs [213,214]. Most infections occur within 6 months of implantation [217] but may also occur several years afterward [218]. Some studies have suggested that early CIED infection (i.e., within 6 months of device implantation) are more likely to be pocket infections, while those >6 months since implantation are more likely to be systemic [219].

In all cases of suspected pocket or systemic infection, blood cultures should be obtained ideally prior to antibiotic administration. Purulent fluid, if present, should be cultured either prior to or during device removal. Chest x-ray, transthoracic and transesophageal echocardiography should also be performed to assess for the presence of septic pulmonary emboli, lead and valvular vegetations, and intracardiac abscesses [51]. Many cases of systemic infection will present with a valvular or lead-associated vegetation seen on echocardiography. However, transthoracic or even transesophageal echocardiography may miss a vegetation despite other clinical evidence suggesting CIED infection [220]. In patients where pocket infection is suspected but clinical manifestations are not overt, 18F-FDG-PET/CT or SPECT/CT can be helpful and may help differentiate pocket infection from other postprocedural CIED complications (e.g., pocket hematoma and post-implantation inflammation) [221].

As mentioned previously, staphylococcal species (e.g., *S. aureus* and coagulase negative staphylococci) are most commonly implicated in CIED-related infections, including both pocket and systemic infections (i.e., device-related endocarditis) [222]. A study of infective endocarditis associated with CIEDs found *S. aureus* and coagulase-negative staphylococci responsible for 60% of cases, whereas enterococci, streptococci, candida, and Gram-negative bacilli combined were responsible for only 22% [223]. Gram-negative bacilli and fungi (e.g., candida) are less often implicated and frequently arise due to a secondary cause [222]. Other studies reported a similar discrepancy between CIED bacteremia caused by *S. aureus* versus Gram-negative bacilli at 54.6% and 12%, respectively [213]. This difference may be explained by properties intrinsic to the culprit pathogen, such as adherence factors and biofilm formation, which are more commonly seen with staphylococcal species [224].

An infectious disease team should be consulted in all cases of CIED infections to help guide the antibiotic selection and course of treatment [51]. Treatment should include empiric antibiotics and evaluation for candidacy for complete device extraction [51]. Device extraction within 3 days of diagnosis has been associated with decreased inpatient mortality and should not be delayed [225]. It should be noted that patients who undergo device extraction for CIED infection are at increased risk for poor outcomes (including death or repeat TLE) compared to patients who undergo device extraction for other reasons [226]. The presence of valvular vegetations or positive blood cultures have also been independently associated with risk of death or repeat device extraction [226], so these patients should be closely monitored after device extraction. Initial antimicrobial coverage should include intravenous vancomycin in addition to empiric broad-spectrum antibiotics while awaiting culture data. It is reasonable to cover Gram-negative bacteria (e.g., cefepime, piperacillin-tazobactam, or carbapenems) in patients with hemodynamic instability or immunocompromised state [227]. The clinical context of the CIED-related infection guides the recommended antibiotic duration. In cases where blood cultures are positive and valvular vegetations are seen, a 4- to 6-week course of antibiotics is warranted. When cultures are positive and vegetations are seen on device leads but not on the valve, 2 to 4 weeks are recommended. Additionally, antibiotics should be given for a minimum of 2 weeks after TLE when *S. aureus* is implicated. In cases where blood cultures are negative, the recommended antimicrobial duration is 2 weeks for pocket infection and 10 days for pocket erosion [51].

After source control has been achieved, the patients’ indication for a CIED re-implantation should be re-evaluated. If still medically necessary, a new CIED should be inserted remotely from the original site (i.e., right pectoral placement if the infected device was placed left pectoral) [51]. Alternatively, a leadless PPM or subcutaneous/extravascular ICD may be considered [51]. While current studies have not shown significant differences in infection frequency between subcutaneous and transvenous CIEDs, recent data suggest lower mortality with subcutaneous versus transvenous device re-implantation following transvenous CIED removal [228]. Acknowledging the optimal time for device re-implantation has not been extensively studied, and the HRS advises postponing new device placement until blood cultures are negative for at least 72 h, assuming a separate infectious source is not present [51].

The prevention of CIED-related infection is essential and CIED-related surgery/intervention should be performed in a controlled, sterile surgical environment and deferred in patients in whom infection is already present [51,229]. As pocket hematomas appear to have the strongest association with CIED infection, heparin products should be avoided periprocedurally (i.e., 24 h pre and 24–72 h post surgery). Vitamin K antagonist anticoagulants should not be bridged, and INR should be reduced to <3.5. Direct acting oral anticoagulants (DOACs) should also be held the day of the procedure. Concurrent antiplatelet use (particularly P2Y12 inhibitors) has been shown to double hematoma formation risk and should be stopped 5–10 days before surgery if possible [229].

Regarding prophylactic antibiotics, systemic intravenous antibiotic prophylaxis is recommended at the time of CIED placement. Antimicrobial selection is based on commonly implicated organisms (as described above) and usually consists of cefazolin or clindamycin (in case of penicillin allergy) for coagulase-negative staphylococci, or alternatively vancomycin if concern exists for methicillin-resistant staphylococci [51]. Recently, the WRAP-IT trial by Tarakji and colleagues (2019) demonstrated a reduction in major CIED-related infection with the use of an absorbable antimicrobial ‘envelope’ at the time of device implantation [230]. This entails placing the device generator in a mesh saturated with rifampin and minocycline during the procedure, leading to the gradual absorption of antibiotics in the device pocket. This randomized trial of nearly 7000 patients showed a marked reduction in the rate of major CIED infection at 12 months when the envelope was used alongside conventional antibiotic prophylaxis [230]. The use of the antimicrobial envelope for CIED infection prevention was also supported in a subsequent metanalysis of ~11,900 patients across six studies, which showed a significant reduction in CIED-related infections in patients who received antibiotic envelopes (RR 0.34; *p* = 0.02) [231]. There are no compelling data that support post-procedural antibiotics for the prevention of CIED infection or their recommendation [51].

## 5. Complications of Subcutaneous ICDs

### 5.1. Inappropriate Device Shocks

One final CIED that merits discussion is subcutaneous ICDs (S-ICDs), which are defibrillators that are implanted entirely subcutaneously and avoid any venous access [5]. Given this difference in device placement, the complications and management of S-ICDs differ from those of traditional transvenous ICDs (TV-ICDs). Inappropriate shocks may occur with S-ICDs, and these are the most common complication associated with S-ICD placement. A 2020 analysis of the FDA’s Manufacturer and User Facility Device Experience (MAUDE) database (detailing 2097 reported S-ICD-related adverse events from February 2016 to February 2018) found that inappropriate shocks constituted 63% of all secondary complications [232]. Similarly to transvenous ICDs, these were most frequently due to device oversensing. As with transvenous devices, T-wave oversensing was implicated in many cases. However, several uncommon causes specific to S-ICDs contributed to oversensing, including device–device interaction from the subcutaneous electrode placement of the S-ICD (e.g., interaction between the electrode and a sternotomy wire) and air release from the device pocket. System migration was also frequently reported as a cause of inappropriate S-ICD shocks, though it was uncertain how operator error may have contributed. Patient factors may contribute to system migration and subsequent inappropriate shocks given that significant weight fluctuations were reported in 10 cases of system migration; however, further studies are needed to clarify this association. As with TV-ICDs, reprogramming is effective for mitigating the risk of inappropriate shock recurrence for S-ICDs [233].

### 5.2. Infection

S-ICDs are preferred over TV-ICDs in patient populations with high risk of infection, as outlined in the 2017 AHA/ACC/HRS guidelines [5]. The PRAETORIAN trial (the first randomized control trial comparing S-ICDs and TV-ICDs in a general ICD population) ultimately showed that S-ICDs were non-inferior regarding device-related complications and inappropriate shocks [234]. A 2022 secondary analysis of this trial found a higher rate of systemic infection during follow-up in those undergoing TV-ICD implant versus S-ICDs (1.2 versus 0%) and lower infection severity in the S-ICD population [235]. While four patients in the S-ICD arm developed an infectious complication during the 4-year median follow-up, these events were adequately treated with oral antibiotics. This may be explained by the absence of endovascular components with S-ICDs. The recent analysis of the MAUDE database by Zeitler et al. [232] found that infectious complications constituted 521/1604 (31%) of all reported adverse events during the study’s 2-year period from February 2016 to 2018, showing that infection remains an important complication of S-ICD implant. However, nearly a quarter of these cases were managed without device extraction, suggesting that many of these cases could be managed non-invasively and may not have been as severe [232].

At present, there are no formal clinical trial data or society guidelines specific to the optimal management of S-ICD infectious complications. Baddour et al. [236] proposed a general approach for the diagnosis and management of suspected S-ICD. Similarly to other CIED or TV-ICD infections, S-ICD pocket infections present with swelling, drainage, and erythema around the implant site. Infectious signs or symptoms tracking along the subcutaneous lead track are unique to S-ICD infections and may be indicative of lead infection. Of note, systemic infection is rare with S-ICDs given the absence of endovascular leads; thus, blood cultures may not be helpful for diagnosing S-ICD infection as these infections tend to localize to the subcutaneous tissue around the device site. While nonspecific, obtaining a complete blood count with differential and inflammatory markers can aid in the diagnosis. Unless purulent drainage can be cultured and/or the device is removed, a causative organism is difficult to identify in the early postoperative period following implantation. While there are no definitive recommendations regarding imaging specifically for identifying S-ICD infections, modalities such as 18F-FDG PET/CT and ultrasound can be helpful when the diagnosis is in question. If the device is extracted, pocket tissue and the extracted ICD should be cultured [236].

Baddour and colleagues suggested that the management of S-ICD infections be guided early (≤30 days following placement) versus late (>30 days following placement), especially as etiologies other than S-ICD infection (e.g., cellulitis and stitch abscesses) can lead to inflammatory changes at the device site [236]. Oral antibiotics (e.g., cephalexin) based on institution-specific antibiograms can be used for the empiric treatment of suspected S-ICD infection for 7–10 days in early presentations where infection is not obvious; patients can be monitored closely for the resolution of local infectious symptoms around the implant area. Empiric oral therapy may be a reasonable initial step in cases where S-ICD infection is not definite given that S-ICD-associated systemic infection is rare. If patients clinically deteriorate or the local implant site changes despite empiric treatment, complete S-ICD removal should be pursued, and an infectious disease consultation should be pursued early in the patient’s course. Device and deep tissue cultures should be taken to identify a culprit organism and screen for antibiotic susceptibilities. In these cases, antibiotics should be extended for a 10–14 day duration and be given either parenterally or orally. The management of late S-ICD presentations is less clear given the scarcity of data regarding classic clinical factors suggesting late S-ICD infection, though device erosion can be observed, and in these cases, the entire device should be removed. Superficial skin changes suggesting infection are less obvious in the late period, and in these cases, systemic infection should be strongly considered, especially if patients develop signs implying bacteremia (chills, fever, and distributive shock). Imaging may be more helpful to aid the diagnosis of late infections [236].

When S-ICD system infection is diagnosed, both the lead and generator should be removed for definitive source control. In clinically stable patients, elective admission and removal within 2–3 days may be appropriate, and the first dose of intravenous antibiotics is delayed until device and tissue cultures have been sent. Emergent removal and empiric antibiotics may be necessary in unstable patients. Intravenous vancomycin is an appropriate first choice as staphylococcal species cause the majority of CIED infections. As with device removal for other instances of CIED infection, the patient should be re-evaluated as to whether a new device is needed. In these cases, the patient should be evaluated for a wearable defibrillator and seen by an infectious disease specialist within 6 weeks after the removal to determine the persistent clearance of infection and the timing of a new device implant. Although uncommon, the re-implantation of an S-ICD after systemic S-ICD infection requiring complete device removal has been reported previously in the literature [237].

### 5.3. Pocket Hematoma

Finally, pocket hematomas represent an important complication of S-ICD placement [232,235]. In contrast with the above analysis of the MAUDE database, the aforementioned secondary analysis of the PRAETORIAN trial by Knops et al. showed that pocket bleeding was the most common complication of S-ICD placement and occurred with greater frequency in recipients of S-ICDs relative to TV-ICDs [234,235]. This may be explained by multiple factors, including a larger pocket being necessary for S-ICD implantation, pressure bandages being used less frequently after S-ICD placement, and lack of standardization regarding periprocedural anticoagulation management for S-ICD placement relative to TV-ICDs [238]. Additionally, a retrospective study of 200 patients from 2018 found lower rates of pocket hematoma when oral anticoagulation and combination antiplatelet therapy with clopidogrel was interrupted without bridging [239]. This investigation implies lower risk of pocket bleeding after S-ICD implant when anticoagulation is interrupted without bridging, though further studies are needed. In contrast with inappropriate shocks and infection, pocket hematoma is observed more frequently as a complication of S-ICD relative to TV-ICD placement but may be remedied with greater implant experience [240].

## 6. Psychological Impact of CIEDs

Despite their effectiveness in treating life-threatening arrhythmias and improving mortality, living with these devices has been shown to have a significant psychological impact on patients. A prior review by Magyar-Russell et al. showed that symptoms of anxiety and depression in patients with ICDs vary widely (8–63% and 5–41%, respectively) [241]. More recent studies have redemonstrated that the rates of these comorbid mental health conditions remain high among patients with ICDs, with rates of anxiety, depression, and post-traumatic stress disorder (PTSD) at 22.6%, 15.4%, and 12.4%, respectively [242]. Additionally, patients with a history of ICD shocks demonstrated higher rates of anxiety (OR = 3.92, 95%CI = 1.67–9.19) and depression (OR = 1.86, 95%CI = 1.34–2.59) [242].

Risk factors associated with developing these mental health conditions and/or experiencing the worsening of symptoms after implantation are largely related to baseline psychological status as well as sociodemographic factors rather than disease- or device-related risk factors. Younger age, female gender, living alone, higher levels of baseline stress or depression, and more perceived ICD-related constraints/concerns have been associated with higher levels of psychological disturbance [243,244,245]. The number of shocks has shown mixed data, with some studies reporting higher rates of psychological distress with higher numbers of shocks [246,247], whereas other studies have not shown such a relationship [245,248,249,250].

It is also pertinent to assess the patient’s knowledge and attitudes regarding their devices, as these have been shown to also contribute significantly to the psychological impact these devices pose. In a study of 1644 CIED recipients from seven European countries, 90% of patients felt sufficiently informed about the indications for their device [251]. Despite 75% of patients noting improvements in their quality of life following device implantation, 40% still had long-term worries about their device and less than 20% had discussed with their physician or thought about device handling in end-of-life circumstances or end-stage disease [251]. Another recent study by Fumagalli et al. on the Italian CIED population evaluating age-related attitudes, worries, psychological effects, and needs showed that younger patients (≤75 years old) experienced more difficulties in their private and professional lives due to their devices, feeling that their life was limited by their device [252]. Additionally, younger patients preferred to be better informed about CIED-related consequences on physical and sexual activities, psychological impacts, and driving limitations compared to older patients [252].

It is critical to understand the psychological impact these devices may have on patients, and identifying those at the highest risk for psychological distress related to their devices with appropriate referral for treatment is essential for achieving maximal gains in quality of life.

## 7. Conclusions

CIEDs are a cornerstone in the treatment of a diverse range of cardiac conditions, with the numbers of implanted devices expected to increase. As these devices are continually undergoing development and innovation, the increased longevity and implant duration means clinicians must remain vigilant of the long-term complications these devices pose to patients. Understanding the patient-specific and non-modifiable risk factors that place patients at increased risk for these complications will allow for careful risk–benefit analysis and improved long-term outcomes. With increasing operator experience and emerging technologies such as leadless pacemakers, it is the hope that future devices and techniques will further mitigate or eliminate many of the difficulties CIEDs present in their current iterations. Initial long-term data are promising, with significant reductions in the rate of complications and device infections at 5-year follow-up [253]. It will be exciting to see how these technologies further advance patient care while minimizing harm.

## Figures and Tables

**Table 2:** Risk factors associated with pacemaker-induced cardiomyopathy.

Risk Factor	Univariate (95% CI)	*p*-Value	Multivariate (95% CI)	*p*-Value	Paper
Age (per 1 year increase)					
	1.01 (0.99–1.02)	0.09			Somma et al. 2022 [142]
	1.00 (0.98–1.02)	0.9			Kurshid et al. 2014 [138]
BMI (per 1 kg/m^2^ increase)					
	0.99 (0.95–1.03)	0.6			Kurshid et al. 2014 [138]
Male Sex					
	1.23 (1.12–1.35)	<0.001			Somma et al. 2022 [142]
	1.8 (1.13–2.86)	<0.01	1.24 (0.69–2.21)	0.48	Cho et al. 2019 [96]
	2.38 (1.28–4.41)	<0.01	2.15 (1.14–4.02)	0.02	Kurshid et al. 2014 [138]
Baseline QRS duration (per msec increase)					
	1.02 (1.01–1.03)	<0.01			Somma et al. 2022 [142]
	1.01 (1.004–1.016) a	<0.01	1.00 (0.99–1.01) a	0.61	Cho et al. 2019 [96]
	1.03 (1.02–1.05)	<0.001	1.03 (1.01–1.05)	<0.001	Kurshid et al. 2014 [138]
Paced QRS duration (per msec increase)					
	1.02 (1.01–1.03)	<0.001			Somma et al. 2022 [142]
	1.013 (1.008–1.019) a	<0.001	1.01 (1.00–1.02) a	0.03	Cho et al. 2019 [96]
	1.01 (0.99–1.03)	0.3	1.01 (0.99–1.03)		Kurshid et al. 2014 [138]
Baseline EF					
	0.95 (0.93–0.97)	<0.001			Somma et al. 2022 [142]
	0.96 (0.94–0.98)	<0.01	0.98 (0.90–1.07)	0.001	Cho et al. 2019 [96]
	0.97 (0.94–1.00)	0.05	0.97 (0.95–1.00)	0.09	Kurshid et al. 2014 [138]
Ventricular pacing frequency (per 1% increase)					
	1.02 (1.01–1.02)	<0.001			Somma et al. 2022 [142]
	1.02 (1.01–1.03)	<0.001	1.01 (1.00–1.02)	0.02	Cho et al. 2019 [96]
	1.00 (0.99–1.02)	0.7			Kurshid et al. 2014 [138]
Atrial Fibrillation					
	1.32 (1.23–1.42)	<0.001			Somma et al. 2022 [142]
	0.97 (0.56–1.69)	0.9			Kurshid et al. 2014 [138]
History of MI					
	1.81 (1.54–2.12)	<0.001			Somma et al. 2022 [142]
	3.2 (1.17–8.78)	0.02	3.31 (0.94–11.7)	0.06	Cho et al. 2019 [96]
CKD					
	1.66 (1.32–2.01)	<0.001			Somma et al. 2022 [142]
Diabetes					
	0.86 (0.69–1.06)	0.14			Somma et al. 2022 [142]
	1.28 (0.68–1.24)	0.8			Kurshid et al. 2014 [138]

Hazard ratios reported for data from Somma et al. 2022; odds ratios reported for data from Cho et al. 2019 and Kurshid et al. 2014. (a) Modified odds ratio to reflect QRS per 1 ms increase (originally reported per 10 ms increase). Abbreviations: BMI (body mass index); EF (ejection fraction); MI (myocardial infarction); CKD (chronic kidney disease).

**Table 3:** Risk factors associated with CIED infection.

Risk Factor	Number of Studies (N)	Pooled Effect	*p*-Value
**Host-related**			
COPD	03	3.35 [1.73, 6.50]	<0.01
CHF	6	1.60 [1.08, 2.36]	0.02
Diabetes mellitus	12	2.00 [1.52, 2.64]	<0.01
ESRD ^a^	8	6.27 [2.86, 13.75]	<0.01
Malignancy	6	2.23 [1.26, 3.95]	<0.01
Renal insufficiency ^b^	5	2.64 [1.17, 5.97]	0.02
Corticosteroid use	7	5.28 [2.29, 12.18]	<0.01
History of device infection	4	7.84 [1.94, 31.60]	<0.01
**Procedure-related**			
Antibiotic prophylaxis used	15	0.37 [0.21, 0.64]	<0.01
Device revision/upgrade	17	2.00 [1.39, 2.89]	<0.01
Generator change	16	1.74 [1.15, 2.63]	<0.01
Hematoma development	8	9.78 [3.70, 25.84]	<0.01
Lead dislodgment/repositioning	4	8.71 [3.86, 19.64]	<0.01
Temporary pacing	7	2.05 [1.02, 4.12]	0.04
<100 procedures by operator	2	2.85 [1.23, 6.58]	0.01
**Device-related**			
Dual-chamber device	10	1.70 [1.11, 2.59]	0.01
Presence of epicardial leads	3	8.09 [3.46, 18.92]	<0.01

^a^. GFR ≤ 15 mL/min or hemodialysis or peritoneal dialysis; ^b^. Glomerular filtration rate or creatinine clearance < 60 mL/min. Abbreviations: COPD (chronic obstructive pulmonary disesase); CHF (congestive heart failure); ESRD (end stage renal disease).

## Abbreviations

The following abbreviations are used in this manuscript:

AI	Aortic insufficiency.
ARVC	Arrhythmogenic right ventricular cardiomyopathy.
AV	Atrioventricular.
AVP	Axillary vein puncture.
BMI	Body mass index.
CHF	Congestive heart failure.
CIED	Cardiac implantable electronic device.
CKD	Chronic kidney disease.
COPD	Chronic obstructive pulmonary disease.
CRT	Cardiac resynchronization therapy.
CRT-D	Cardiac resynchronization therapy defibrillator.
CRT-P	Cardiac resynchronization therapy pacemaker.
CSP	Conduction system pacing.
CVC	Cephalic vein cutdown.
CVO	Central venous obstruction.
CVS	Central venous stenosis.
DM	Diabetes mellitus.
ESRD	End-stage renal disease.
HCM	Hypertrophic cardiomyopathy.
HV	High-voltage.
ICD	Implantable cardioverter–defibrillator.
ILR	Implantable loop recorder.
IVF	Idiopathic ventricular fibrillation.
IVUS	Intravascular ultrasound.
LBBB	Left bundle branch block.
LBBP	Left bundle branch pacing.
LIA	Lead Integrity Alert.
LL-PPM	Leadless pacemaker.
LNA	Lead Noise Algorithm.
LRL	Lower rate limit.
LRVO	Lead-related venous obstruction.
MI	Myocardial infarction.
PED	Primary electrical disease.
PiCM	Pacemaker-induced cardiomyopathy.
PMT	Pacemaker-mediated tachycardia.
PPM	Permanent pacemaker.
PTSD	Post-traumatic stress disorder.
PVARP	Post-ventricular atrial refractory period.
PVC	Premature ventricular contraction.
RA	Right atrium.
RBB	Right bundle branch.
RBP	Right bundle pacing.
RNRVAS	Repetitive non-reentrant ventriculoatrial synchrony.
RV	Right ventricle.
S-ICD	Subcutaneous ICD.
SVC	Superior vena cava.
SVP	Subclavian vein puncture.
TV-ICD	Traditional transvenous ICD.
TVP	Transvenous lead extraction.
TR	Tricuspid regurgitation.
VA	Ventriculoatrial.
VF	Ventricular fibrillation.
VT	Ventricular tachycardia.
VUS	Venous duplex ultrasound.
